# Hybrid magnetron sputtering of ceramic superlattices for application in a next generation of combustion engines

**DOI:** 10.1038/s41598-022-06131-9

**Published:** 2022-02-11

**Authors:** Bruno César Noronha Marques de Castilho, Alisson Mendes Rodrigues, Pedro Renato Tavares Avila, Raíra Chefer Apolinario, Tamires de Souza Nossa, Magdalena Walczak, Jucielle Veras Fernandes, Romualdo Rodrigues Menezes, Gelmires de Araújo Neves, Haroldo Cavalcanti Pinto

**Affiliations:** 1 São Carlos School of Engineering (EESC), University of São Paulo (USP), São Carlos, SP CEP: 13563-120 Brazil; 2 Laboratory of Materials Technology (LTM), Department of Materials Engineering, Federal University of Campina Grande (UFCG), Campina Grande, 58429-900 Brazil; 3 Laboratory of Materials (LabMat), Federal Institute of Education, Science and Technology of São Paulo (IFSP), Itapetininga, 18.202-000 Brazil; 4 Department of Mechanical and Metallurgical Engineering, Escuela de Ingeniería, Pontificia Universidad Católica de Chile, Vicuña Mackenna, 4860 Santiago, Chile

**Keywords:** Engineering, Materials science

## Abstract

A hybrid magnetron sputtering process (dcMS/HiPIMS) was developed to manufacture nanostructured CrN/Cr_1-x_Al_x_N multilayers, motivated by improving the low-emission efficiency when applied on gas-nitrided diesel piston rings of a next-generation of combustion engines. In order to improve the mechanical, tribological, and corrosion behavior of the multilayers, the hybrid dcMS/HiPIMS process was designed by selecting the optimal sputtering procedure applied to AISI 440 base steel. The effect of substrate bias and carousel rotational speed on the phase composition, crystallographic texture, residual stresses, surface roughness, coating periodicity and densification, instrumented hardness, elastic modulus, as well as wear and corrosion resistance was determined. The results have demonstrated that hybrid magnetron sputtering produces multilayers with a superlattice structure, which outperforms commercial PVD coatings of CrN for diesel piston rings manufactured by cathodic arc evaporation. Also, multilayer periodicities in the range of 5 to 10 nm yield the best tribological performance under bench tests for the piston ring/cylinder liner system.

## Introduction

Nowadays, environmental protection is one of the most immediate and indispensable humankind’s issues. Internal combustion engines are at the center of this discussion since the consumption of fossil fuels, in particular petroleum diesel, results in the generation of nitrogen oxides (NOx), hydrocarbons and particulate matter, which contribute to the greenhouse effect, thus deteriorating air quality and thereby putting the health of humankind at risk^1,2^.

The European Union (EU) was one of the first regions to implement the control of combustion engine emissions. In 1992, the EURO 1, i.e., the first attempt to implement such regulations, defined limits for the emission of heavy-duty diesel engines, and since then, year-on-year, the regulations were getting stricter. In 2017, for instance, the European Union proposed diminishing the NOx content in the emissions of diesel engines from 0.50 to 0.080 g/kWh^3,4^.

However, to achieve expressive gains concerning cleaner air and low emissions, the mechanical components of the power cell need to be manufactured from materials that decrease friction losses and diminish wear. The combustion process also has to be optimized owing to the use of cleaner fuels with reduced sulfur content and, as a result, lower pollutant generation. Lower sulfur content negatively affects the lubricity of combustion residues, thus demanding novel coatings to minimize wear and fuel consumption while maintaining low friction in adverse working conditions.

In the design of internal combustion engines, the piston ring pack is the most technically challenged sub-system regarding tribological issues due to higher wear ratio and scuffing^5^. One of the main objectives of piston rings is to seal the combustion chamber, and as the piston ring is worn out, more oil from the engine will be burned in the combustion chamber, thus raising the vehicle's pollutant emission levels. Indeed, more than half of the engine’s oil consumption in diesel-powered automobiles comes from the piston ring pack^6^.

The use of coatings is a suitable solution to extend the lifetime of piston rings. However, a broad range of materials and deposition processes may be considered in manufacturing these piston rings. In terms of materials, the most important alternatives include binary and ternary nitride-based coatings. Among them stands out the chromium nitride (CrN) and chromium aluminum nitride (Cr_1-x_Al_x_N). Both have exhibited higher thermal stability and corrosion resistance when compared to titanium nitride (TiN) or titanium aluminum nitride (TiAlN)^7^. Also, the possibility of alternately depositing such nitrides has shown that nanostructured multilayers may be achieved with improved mechanical and tribological properties compared to single layers^8–10^.

Physical Vapor Deposition (PVD) is a versatile technology available to manufacture coatings from several material classes (i.e., ceramic^11–13^, metallic^14^, polymeric^15–17^ and composites^14^) with a broad range of properties, such as wear resistance, low friction, corrosion resistance, as well as for optical and electrical applications^18^. Regarding the physical method to produce the vapor phase^19^, the PVD techniques are classified into two main processes: sputtering and evaporation^20^. Cathodic arc evaporation is one of the main representatives of commercial PVD processes and it is often used to manufacture hard coatings for tribological applications due to its high deposition rates^21^. Magnetron sputtering is an alternative that is being considered for the near future since it allows for obtaining superior mechanical properties by enhancing coating quality and integrity^18,20,22^.

Among the magnetron sputtering processes, the direct current magnetron sputtering (dcMS) and high power impulse magnetron sputtering (HiPIMS) stand out. dcMS can be easily implemented due to the low number of parameters to be optimized during the deposition process. In this technique, a continuous current is applied to the metallic target, and permanent magnets are employed to produce the magnetic field used to confine the plasma close to the target^23^. In order to achieve a considerable level of ionization and improve the coating properties, high power levels need to be delivered to the target. However, since dcMS relies on continuous current levels, the metallic target may be overheated, thus causing permanent damage^21^. To overcome this issue, HiPIMS appears as an alternative, which uses elevated peak power and current applied in short pulses^13,19,24–26^. This increases plasma density and the ionization of the target, although the deposition rate is lower than in dcMS^24,27^. Coatings manufactured by HiPIMS have shown to be denser while having excellent surface finish and adhesion to the substrate^28^.

The combination of dcMS and HiPIMS in a hybrid process offers the possibility of achieving higher deposition rates than pure HiPIMS, while allowing for the production of nanostructured multilayers with superlattice structures by tailoring the way how different target materials are switched to the different power supplies. Bobzin et al.^29^ deposited (Cr, Al)N coatings using dcMS, HiPIMS and a hybrid dcMS/HiPIMS. The authors used two cathodes, one connected to a dcMS and another connected to the HiPIMS, and both using a Cr target with aluminum plugs. The coatings resulting from the hybrid process showed a gain in deposition rate compared to pure HiPIMS due to the presence of the dcMS, while hardness and elastic modulus of the hybrid assumed intermediate values between HiPIMS and dcMS^29^.

In another study, Paulitsch et al.^30^ deposited CrN using a pure HiPIMS, a pure dcMS and a hybrid dcMS/HiPIMS and they have shown that the deposition rate increases and the hardness decreases when the applied power in the dcMS is increased for the hybrid configuration. Meanwhile, Hovsepian et al.^31^ deposited a multilayered coating with nanolayers of TiAlCN/VCN and observed reduced residual stresses, high hardness and extended tool life when depositing with a hybrid HiPIMS/dcMS system. Kamath et al.^32^ also successfully deposited a multilayered TiAlCN/VCN coating using a combination of HiPIMS and dcMS, observing gains in the deposition rate compared with pure HiPIMS.

The periodicity of the superlattice can be adjusted by changing the carrousel speed, which in turn can often influence coating properties, such as hardness, fracture toughness and corrosion resistance^33–37^. Another way of improving coating properties is by increasing the applied substrate bias, which is often observed to increase both hardness and residual stresses of the coatings up to a certain limit (for both dcMS and HiPIMS)^38,39^.

Hence, in the present study, we propose to combine dcMS and HiPIMS in a hybrid magnetron sputtering process to manufacture CrN/Cr_1-x_Al_x_N multilayers with a superlattice structure. The effect of substrate bias on the mechanical and tribological behavior of the coating was evaluated, while the superlattice periodicity was optimized by changing the carrousel speed. This methodology can be applied to the next generation of combustion engines to improve the mechanical, tribological, and corrosion properties of coatings for diesel piston rings, while maintaining adequate deposition rates and productivity. The results are compared to experimental wear data acquired from commercial CrN coatings used in diesel piston rings and manufactured by arc evaporation.

## Results and discussions

### Effect of substrate bias

**Figure 1:**
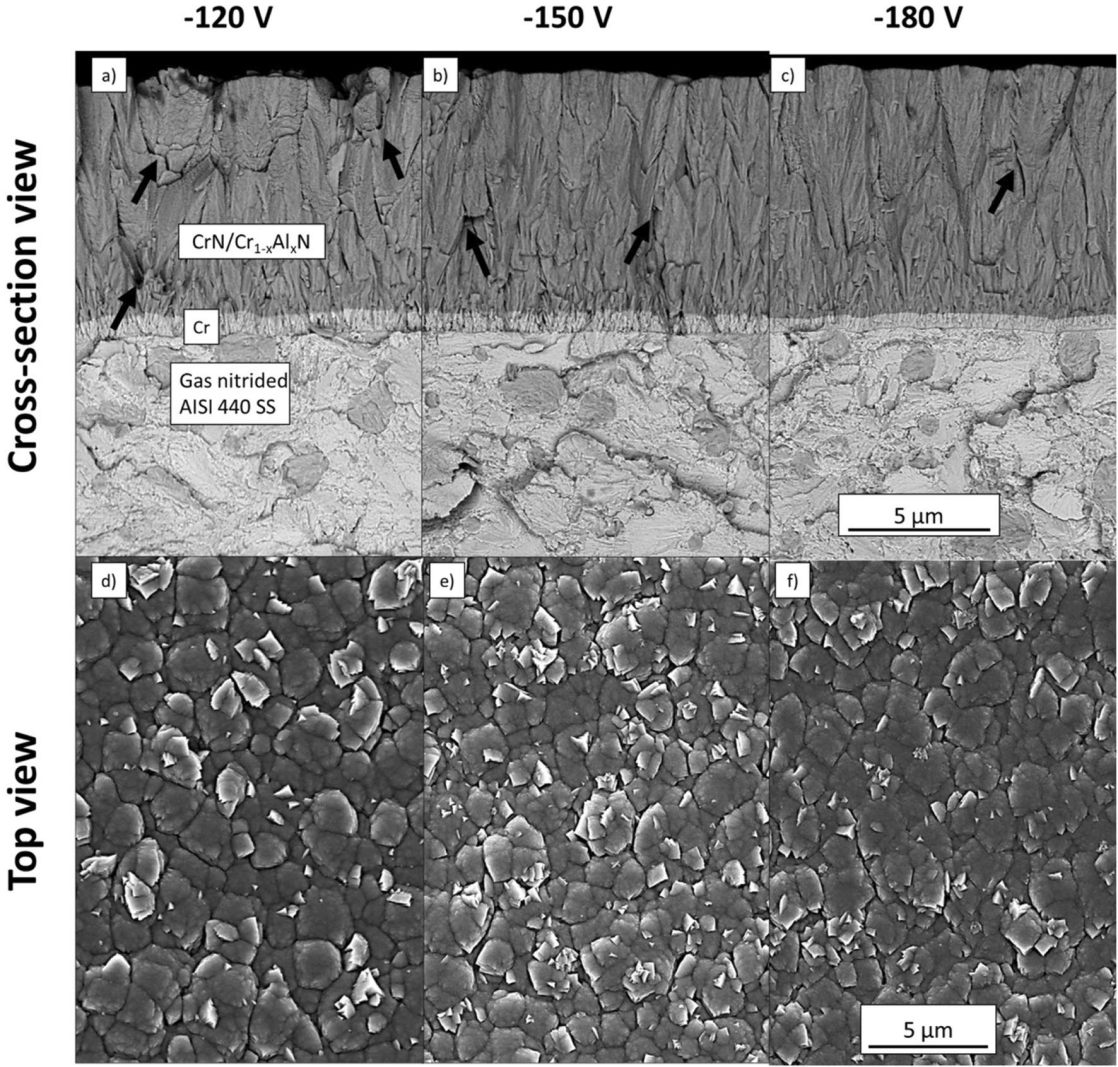
FEG–SEM micrographs of fractured cross-sections (**a**–**c**) and top surfaces (**d**–**f**) observed from CrN/Cr_1-x_Al_x_N multilayers manufactured at constant carousel speed (1 rpm) and the different biases. The black arrows indicate the presence of intergranular porosity.

Figure 1a–f show FEG-SEM micrographs acquired from the fractured cross-section and the top surface of the CrN/Cr_1-x_Al_x_N multilayers manufactured at constant carousel rotational speed of 1 rpm and different bias (− 120 V, − 150 V, or − 180 V). From the fractured cross-sections (Fig. 1a–c), the effect of bias applied to the piston rings on the densification of the coating is apparent since intergranular porosity decreases as the bias increases. This trend is also present on the top surface, as shown in Fig. 1d–f. The highest coating density is achieved for a bias of − 180 V owing to the increased kinetic energy of the ions impinging on the coating surface for more negative levels of bias. These results agree with the previous experimental^28^ and theoretical studies^40^. The more negative bias presented no significant impact in the deposition rate of the coatings, calculated to be around 0.42 µm/h for all samples. This effect is visible in Fig. 1a–c, where all coatings have a similar thickness. The top surfaces reveal that the grain morphology is not affected by the bias level.

**Figure 2:**
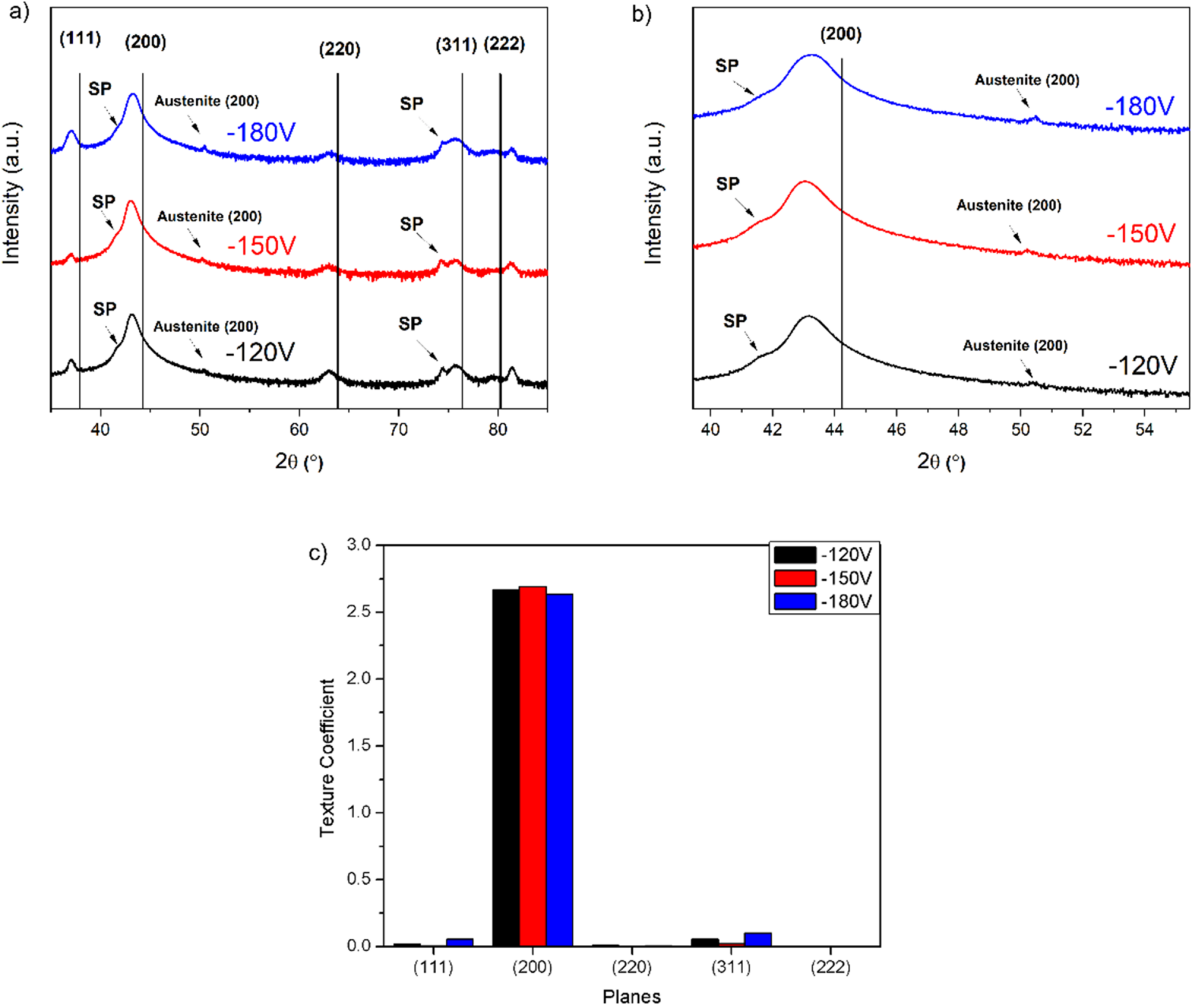
X-ray diffractograms (**a**), detail around the CrN (200) peak position showing the satellite peaks (**b**) and texture coefficients (**c**) for the CrN/Cr_1-x_Al_x_N multilayers produced at constant carousel speed (1 rpm), using different bias levels (− 120 V, − 150 V or − 180 V). The satellite peaks (SP) indicate the formation of a superlattice. Austenite peaks from the substrate are also indicated.

Figure 2a,b shows X-ray diffractograms and the correspondent texture coefficients for the CrN/Cr_1-x_Al_x_N multilayers manufactured at constant carrousel rotational speed (1 rpm) and three different bias levels. Independent of the bias condition, the only phase formed in the coatings is the face-centered cubic (fcc) CrN (Fig. 2a). The absence of diffraction lines related to hexagonal AIN reveals that Al acts as a substitutional element within the crystalline structure of fcc-CrN. Indeed, as shown elsewhere^41^, the addition of small amounts of Al results in forming a CrAlN metastable ternary solid solution, which exhibits improved mechanical and tribological properties. Further, Fig. 2a reveals the formation of superlattices, where the fcc-structures of CrN and Cr_1-x_Al_x_N grow epitaxially with similar lattice parameters on each other^8^. This superstructure causes in all manufactured coatings the occurrence of satellite peaks around the (311) diffraction line at 2θ ~ 75°^42^, seen in detail in Fig. 2b.

The texture coefficients (see Fig. 2c) indicate in addition that the fcc-structures grow with a strong (100) fiber orientation in the out-of-plane direction, and the substrate bias does not exhibit a considerable impact on this type of preferential orientation of growth.

**Figure 3:**
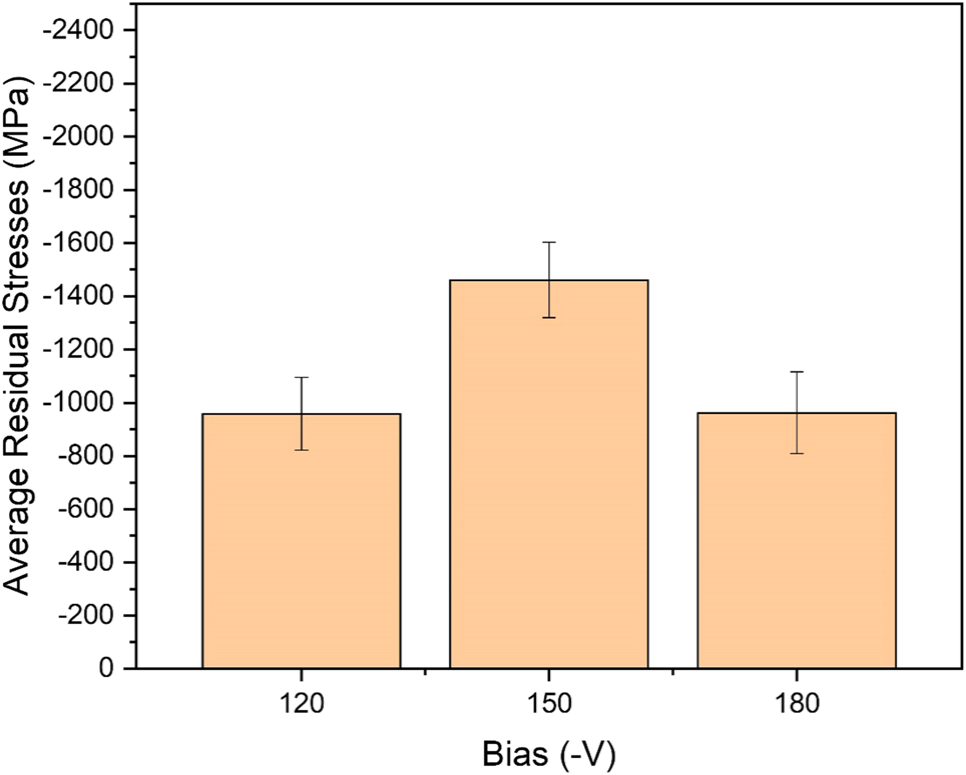
Residual stress evolution in the CrN/Cr_1-x_Al_x_N multilayers deposited onto gas nitrided diesel piston rings at 1 rpm as a function of the bias level.

The mean residual stresses in the CrN/Cr_1-x_Al_x_N multilayers were determined by XRD and the results are displayed in Fig. 3 as a function of the bias applied to the piston ring. Independent of the bias level, expressive compressive stresses (above 1 GPa) are generated in the multilayers. The maximum compression is encountered for intermediate bias, i.e., − 150 V. This confirms that the adequate bias condition must be determined in advance for manufacturing coatings with optimum performance. In addition, too high bias levels seem to cause micro-defects due to the extreme energy of the impinging ions on top of the growing multilayers, thus leading to a certain stress relief compared to the optimum bias level.

**Figure 4:**
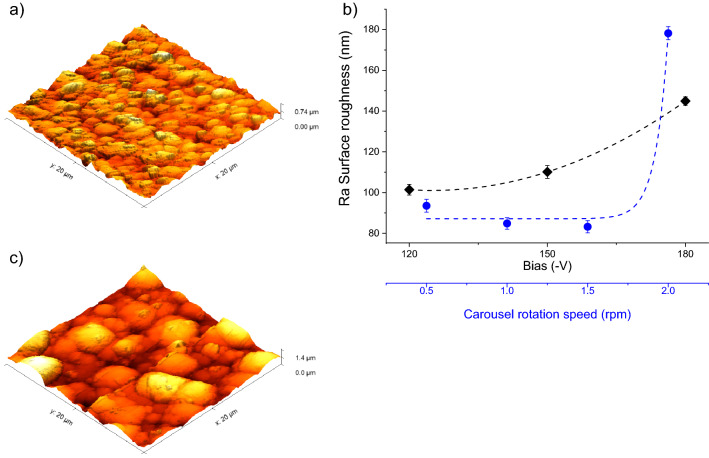
(**a**) 3D AFM surface map obtained from the coating manufactured at 1 rpm and − 150 V, (**b**) effect of bias and carousel rotational speed on the surface roughness and (**c**) 3D AFM surface map obtained from the coating manufactured at 2.0 rpm and − 150 V.

The mean surface roughness (Ra) of the CrN/Cr_1-x_Al_x_N multilayers was evaluated from 3D AFM surface maps. Figure 4a displays a representative 3D AFM surface map acquired from the coating manufactured at 1 rpm and − 150 V. The Ra values determined for the coatings produced at 1 rpm are 101.4 nm, 110.1 nm, and 144.9 nm when using bias of − 120 V, − 150 V, and − 180 V, respectively (Fig. 4b). More elevated substrate bias enhances the energy of the impinging ions on the coating surface, and this generates an increasing number of surface imperfections, such as grains with cauliflower-like morphology, that affect the mean surface roughness of the coatings^22,41,42^. These cauliflower-like structures are usually produced by impurities coming from the chamber and get deposited at the surface of the coating during deposition^43,44^. However, it could also be hypothesized that small clusters of CrN/CrAlN with slightly different orientations could be forming during the deposition and acting as seeds for such defects. Thus, as the carrousel speed increases, the chance of one of these clusters being deposited at the surface increases, significantly increasing the roughness at 2 rpm.

**Figure 5:**
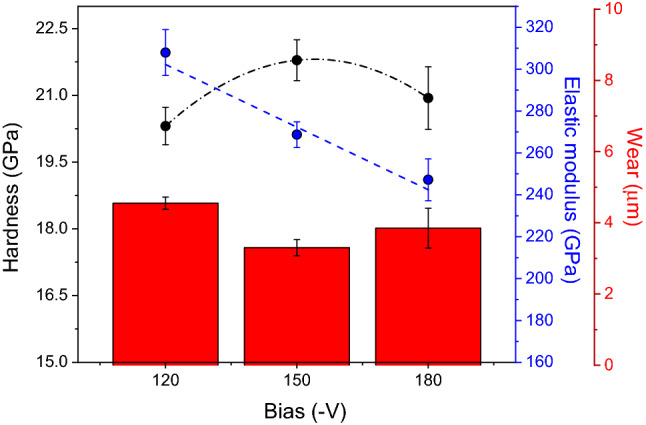
Mechanical properties and wear depth against the cylinder liner of CrN/Cr_1-x_Al_x_N multilayers deposited onto gas nitrided diesel piston rings at 1 rpm with different bias levels (− 120 V, − 150 V, and − 180 V).

The mechanical and tribological properties were characterized with the outlook of applications in combustion engines, where a compromise between mechanical and tribological properties is indispensable. Figure 5 compares hardness (H) and elastic modulus (E) determined from instrumented nanoindentation tests, with the results of wear against the cylinder liner, expressed in wear depth at the end of the test, for all CrN/Cr_1-x_Al_x_N multilayers manufactured at constant carrousel rotational speed (1 rpm) and different bias levels applied to the gas nitrided piston rings (− 120 V, − 150 V, or − 180 V). The hardness values do not show a monotonic trend with bias and yield 20.3 ± 0.4 GPa, 21.8 ± 0.5 GPa, and 20.9 ± 0.7 GPa for the coatings manufactured at − 120 V, − 150 V, and − 180 V, respectively. The hardness values follow the same trend as the compressive residual stresses in the multilayers, i.e., maximum hardness and compression are observed for intermediate bias. This reinforces the expectation that extremely high bias damages coating integrity. There is, however, a tendency of elastic modulus decreasing linearly with bias, thus producing 308.0 ± 11.0 GPa, 268.8 ± 6.1 GPa, and 247.2 ± 10.0 GPa for − 120 V, − 150 V, and − 180 V bias, respectively. This suggests that atomic bonding within the superlattice is impaired by increasing the bias level in a range beyond − 100 V.

In spite of diminishing atomic bonding in the range of bias required in the present study, the wear depths were 4.55 ± 0.50 µm, 3.30 ± 0.60 µm and 3.85 ± 0.56 µm for − 120 V, − 150 V, and − 180 V substrate bias, respectively. From the analysis of Fig. 5, and under the perspective of compressive residual stresses, hardness and wear, the CrN/Cr_1-x_Al_x_N multilayer manufactured at 1 rpm and − 150 V exhibits the best requirements for piston ring applications.

**Figure 6:**
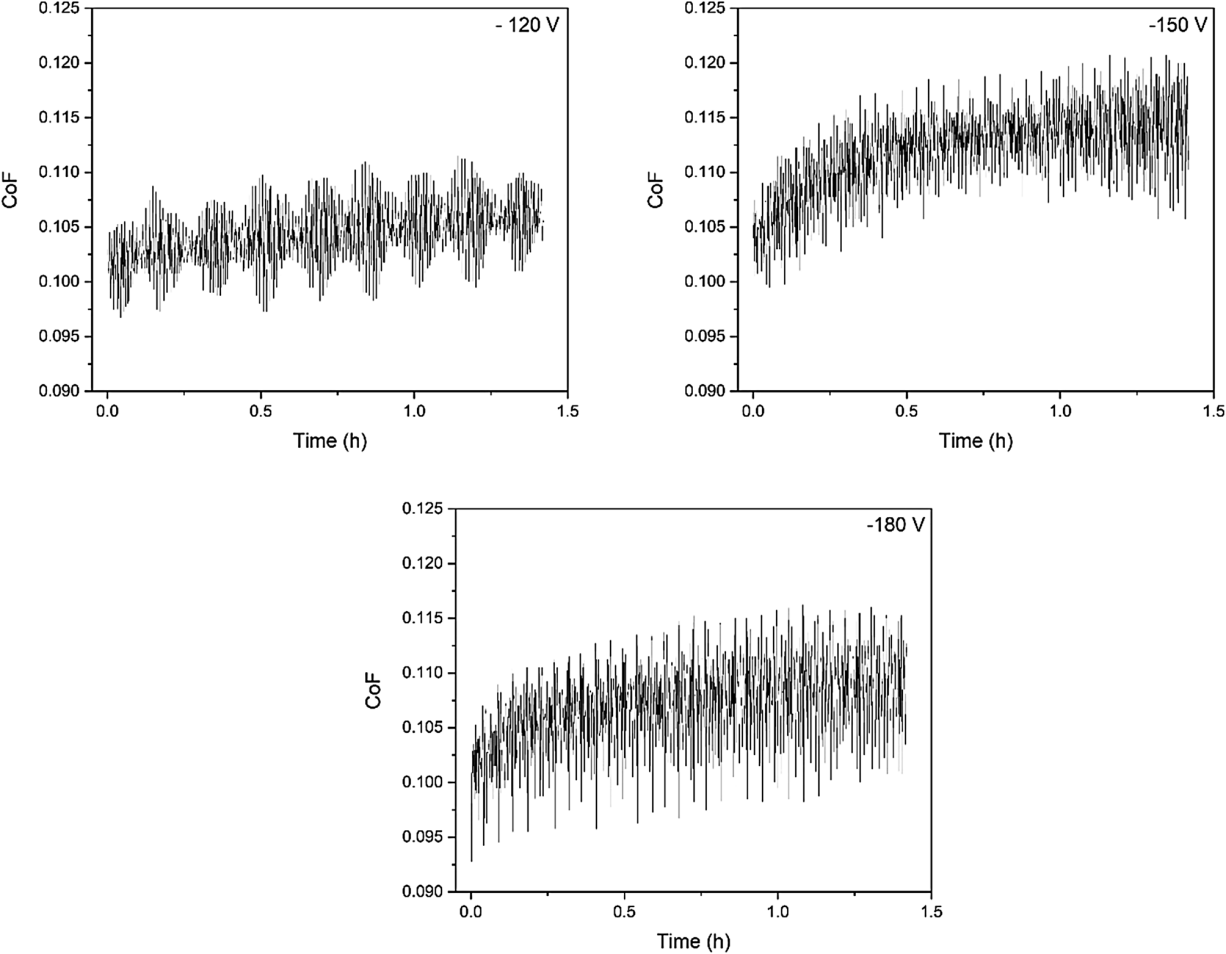
CoF evolution during wear tests for the multilayer coatings produced using different substrate bias potential.

Figure 6 exhibits the evolution of CoF during the wear tests against the cylinder liner for the three bias conditions. There is a running-in period with an increase in the CoF followed by a stabilization around 0.1, mainly for − 150 and − 180 V. The coating produced with − 120 V bias presents the lowest CoF. This may be related to the fact that the lowest bias level associated with the lowest coating integrity favors coating detachment during wear and the loose rolling particles within the contact zone reduce the friction between coating and cylinder liner. Despite this, all conducted wear tests presented values of CoF compatible or inferior to literature values for CrN-based coatings in engine operation^45–49^.

Although the highest value of elastic modulus was not observed for the CrN/Cr_1-x_Al_x_N multilayer manufactured at 1 rpm and − 150 V, the maximum resistance to plastic deformation, as inferred from H^3^/Er^2^, (0.14 GPa, see Table 1) along with the lowest wear against the cylinder liner and surface roughness (Fig. 4b) were determining factors to consider this set of deposition parameters for further process optimization. Therefore, the next section will deal with the effect of multilayer periodicity on the mechanical and tribological properties by modifying the carousel rotation speed for the substrate bias of − 150 V.

**Table 1:** Summary of H, E, wear against the cylinder liner, and H^3^/E^2^ values obtained for CrN/Cr_1-x_Al_x_N multilayers manufactured at constant carousel rotation speed (1 rpm), and different bias levels (− 120 V, − 150 V, and − 180 V).

Property	Bias (−V)		
	120	150	180
H (GPa)	20.3 ± 0.4	21.8 ± 0.5	20.9 ± 0.7
E (GPa)	308.0 ± 11.0	268.8 ± 6.1	247.2 ± 10.0
Wear depth (µm)	4.55 ± 0.50	3.30 ± 0.60	3.85 ± 0.56
H^3^/E^2^ (GPa)	0.09	0.14	0.14
Average COF	0.105 ± 0.004	0.113 ± 0.003	0.108 ± 0.003

### Effect of multilayer periodicity

**Figure 7:**
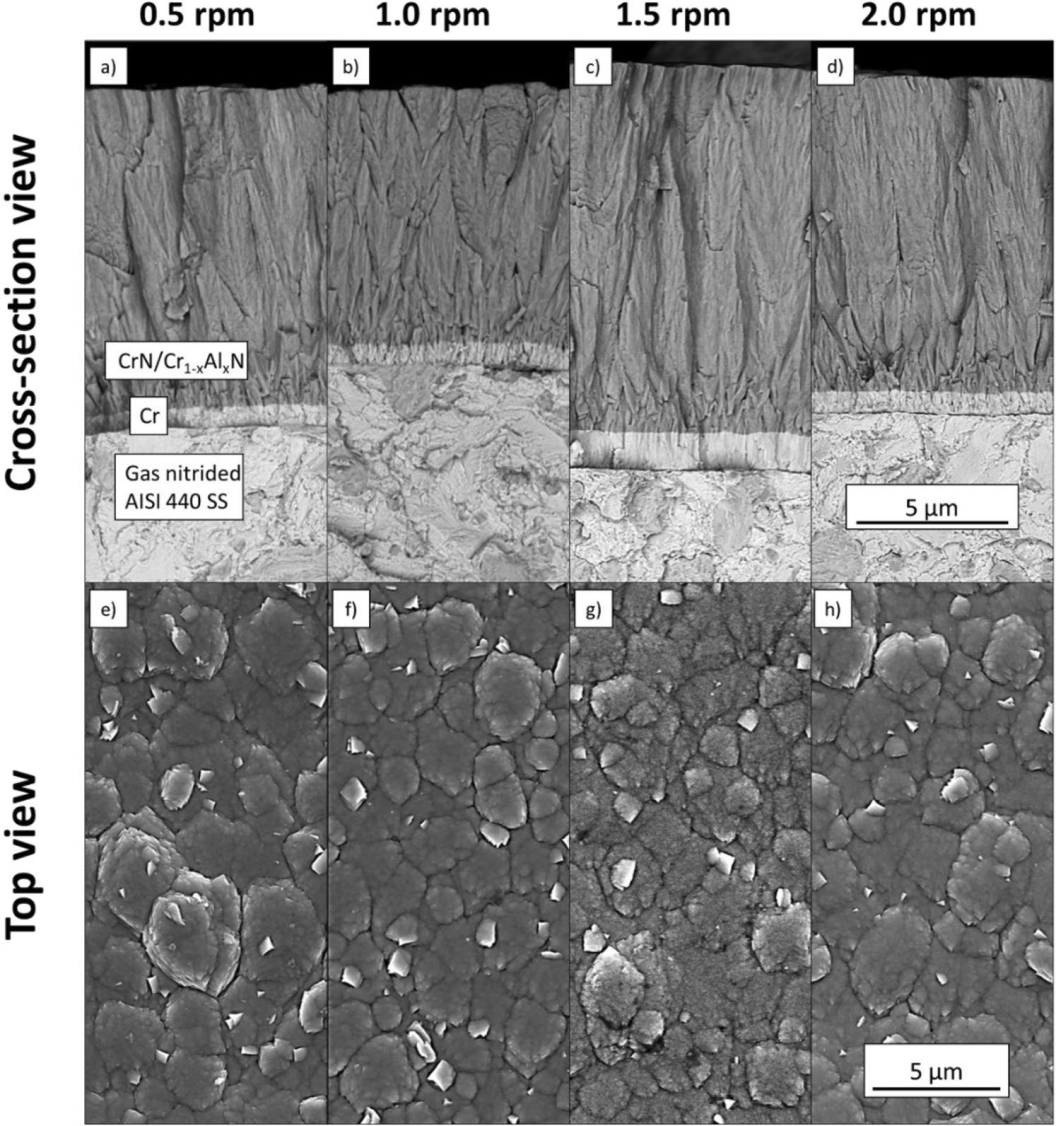
(**a**–**h**) FEG-SEM micrographs of fractured cross-sections (**a**–**d**) and top surfaces (**e**–**h**) acquired from CrN/Cr_1-x_Al_x_N multilayers manufactured at constant substrate bias (− 150 V) and different carousel rotational speeds (0.5 rpm, 1.0 rpm, 1.5 rpm, and 2.0 rpm).

Figure 7a–h displays FEG-SEM micrographs acquired from cross-section and the top surface of the CrN/Cr_1-x_Al_x_N multilayers manufactured at a constant bias (− 150 V) and different carousel rotational speeds (0.5 rpm, 1.0 rpm, 1.5 rpm, and 2.0 rpm). The total thickness (coating + base layer) was measured from Fig. 7a–d and the values obtained were 10.5 ± 0.1 µm, 9.1 ± 0.1 µm, 12.1 ± 0.1 µm and 11.1 ± 0.2 µm for 0.5 rpm, 1.0 rpm, 1.5 rpm and 2 rpm, respectively. The variations in total coating thickness for different carousel rotations caused slightly different sputtering times for each deposition cycle. The deposition rate for all carousel rotational speeds was 0.5 ± 0.1 µm/hour. Also, it was possible to verify that the carousel rotation speed does not significantly affect the grain morphology (see Figs. 1d–f and 7e–h). The deposition rate of the hybrid process is notably lower than the deposition for cathodic arc evaporation observed in the literature, which can reach up to 50–70 µm/hour, depending on material and deposition parameters^50,51^. However, coatings produced by cathodic arc usually present metal inclusions/defects^51^, which are not observed in the current hybrid coating. Furthermore, the proposed hybrid coating has higher hardness and wear resistance which allows for thinner coatings to be produced, which could potentially make it attractive for industrial applications.

**Figure 8:**
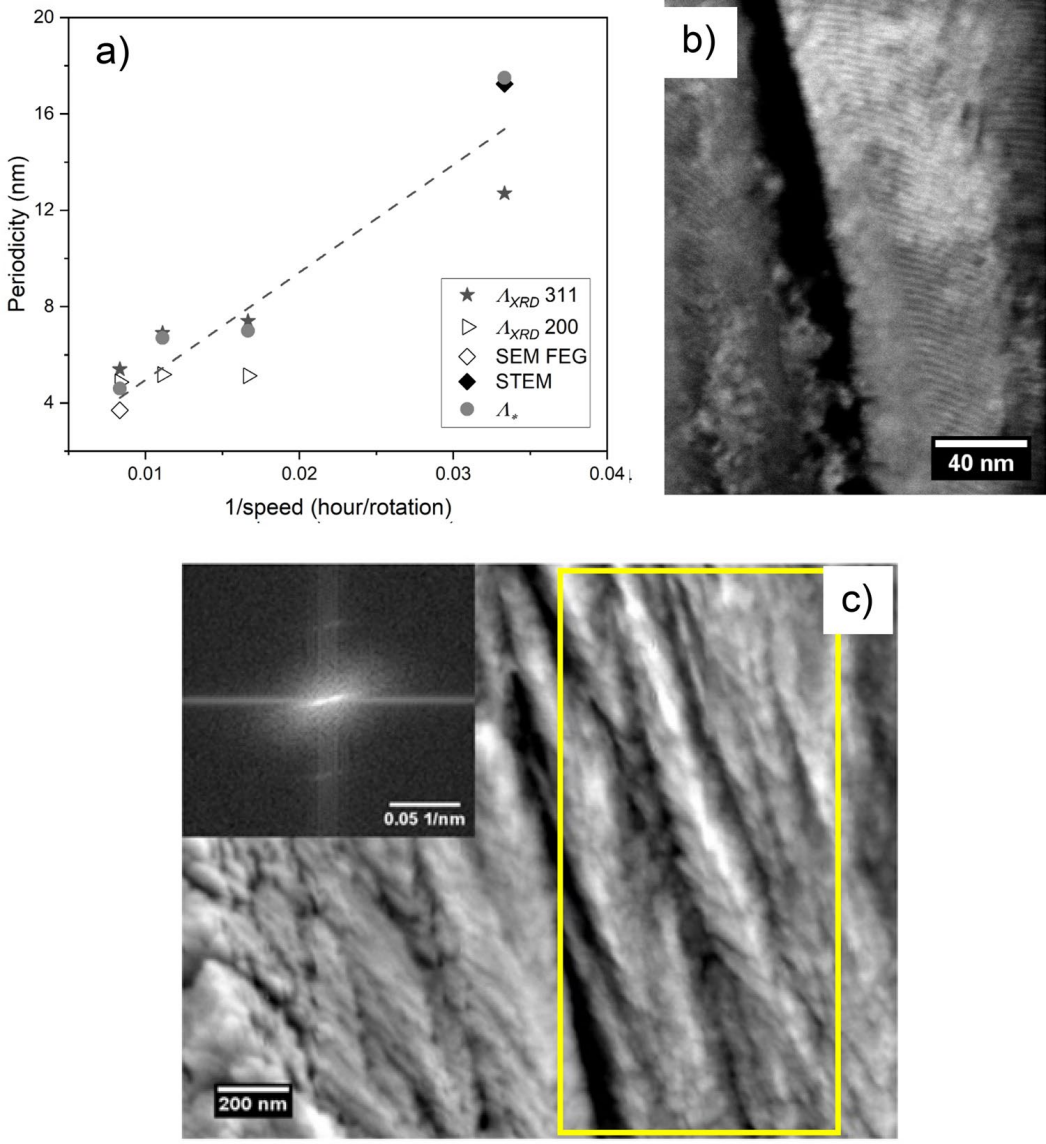
(**a**) Effect of the carousel rotational speed on the multilayer periodicity according to four distinct methodologies for CrN/Cr_1-x_Al_x_N multilayers manufactured at a constant bias level (− 150 V), (**b**) STEM micrograph acquired from the multilayer deposited at − 150 V and 0.5 rpm, and (**c**) High-resolution FEG-SEM micrograph acquired from the cross-section of the multilayer deposited at − 150 V and 2.0 rpm.

Figure 8a shows how the periodicity depends on the inverse of the carrousel rotational speed by applying four distinct methodologies to the CrN/Cr_1-x_Al_x_N multilayers manufactured at constant bias level (− 150 V). The values corresponding to the gray circles were calculated based on Eq. (4) (${\Lambda }_{*}$) and the coating thicknesses required were measured from FEG-SEM micrographs (Fig. 7a–d). In agreement with the literature^52^, the lowest periodicity was observed for coatings manufactured at 2.0 rpm. STEM, FEG-SEM, and XRD were accomplished to verify the formation of the multilayer architecture of the coatings. STEM and FEG-SEM micrographs were acquired from fractured cross-sections of the CrN/Cr_1-x_Al_x_N multilayers grown at − 150 V with 0.5 rpm and 2.0 rpm, respectively. The periodicity value obtained from the STEM micrograph for 0.5 rpm in Fig. 8b was very close to that calculated by Eq. (4), 17.25 nm, and 17.50 nm, respectively. The figure inserted on the left top side of Fig. 8c displays the reciprocal space pattern obtained from the Fast Fourier Transform (FFT) applied to the high-resolution FEG-SEM micrograph, more precisely on the region delimited by a yellow rectangle. The periodicity value obtained for 2.0 rpm rotational speed using the FFT method and its correspondent reciprocal space pattern amounts to 3.7 nm, which is also very close to the valuecalculated using Eq. (4) (4.6 nm). Still considering Fig. 8a, the experimental XRD values are represented by full star points were calculated from XRD (${\Lambda }_{XRD}$) and calculated based on Eq. (3). The XRD periodicities agree well with the values obtained from Eq. (4), FEG-SEM and STEM analyses.

**Figure 9:**
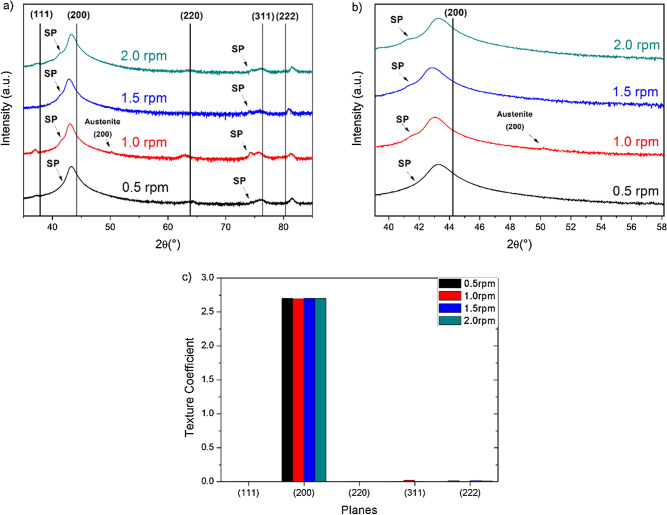
X-ray diffractograms for the CrN/Cr_1-x_Al_x_N multilayers deposited at different rotational speeds show satellite peaks that characterize superlattices (**a**) and in detail in (**b**). Texture coefficients indicate the predominance of (100) fiber texture in the normal surface direction of the multilayers (**c**).

In agreement with the CrN/Cr_1-x_Al_x_N multilayers grown at constant carousel rotational speed (1 rpm) and different bias (− 120 V, − 150 V, and − 180 V), the grain morphology remains nearly unchanged on the top surface (see Figs. 1d–f and 7e–h). As presented in Fig. 9a, the fcc-CrN phase was the sole crystalline phase identified by XRD, and no hcp-AlN diffraction lines were observed. The superlattice structure was confirmed once again through the identification of satellite peaks around the (311) diffraction line at 2θ ~ 75°, as seen in detail in Fig. 9b, whereas the texture coefficient in Fig. 9c revealed that the columnar grains follow preferentially the (100) crystal direction.

**Figure 10:**
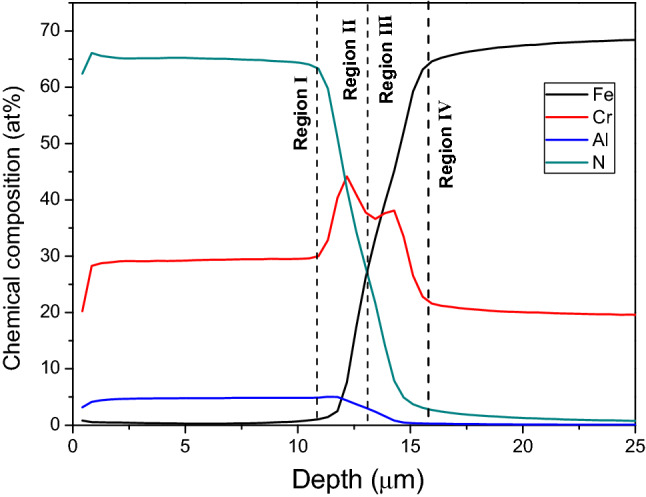
GDOES depth profile of chemical composition across the CrN/Cr_1-x_Al_x_N multilayer and the Cr base layer deposited onto an AISI 304L flat substrate using rotational speed of 1.0 rpm and the bias of − 150 V.

The chemical modulation within the CrN/Cr_1-x_Al_x_N multilayers manufactured with different periodicities was analyzed using the GDOES technique. In general, there was no apparent effect of bias or carousel rotational speed on the compositional depth profiles. Figure 10 exemplifies, therefore, a GDOES depth profile obtained across the CrN/Cr_1-x_Al_x_N multilayer grown on gas nitride piston rings at 1 rpm and − 150 V, in which it is possible to identify four chemical regions. The topmost Region I corresponds to the CrN/Cr_1-x_Al_x_N multilayer. Regions II and IV correspond to the Cr base layer and gas-nitrided AISI 440 steel, respectively. Region III corresponds to the Cr peak (at about 14.33 µm depth) related to a shallow Cr implantation into the gas nitrided AISI 440 steel substrate due to ion etching. One can notice that interfaces between these regions are not abrupt as elements drop continuously when changing from one region to another. This is explained by chemical interdiffusion between the characteristic regions at 400 °C during coating deposition. Owing to the formation of wavy interfaces between CrN and Cr_1-x_Al_x_N sub-layers, as displayed in Fig. 8b, the chemical composition across the coating appears as homogeneous throughout the entire multilayer thickness (~ 10 µm) and does not directly reveal its chemical modulation. However, this can be estimated if we consider that the nitrogen content shall remain nearly the same across the coating when maintaining the N_2_/Ar ratio of the plasma atmosphere. In agreement with our previous investigations^10^, the incorporated nitrogen content in the nitride sub-layers is independent of further process parameters, such as pulse frequency or substrate bias. Hence, by assuming that nitrogen achieves 65 at.% in both sub-layers, the individual composition of each nitride can be given as follows: Cr_0.35_N_0.65_ and Cr_0.254_Al_0.096_N_0.65_ (i.e., Al/Cr ratio of 0.38).

**Figure 11:**
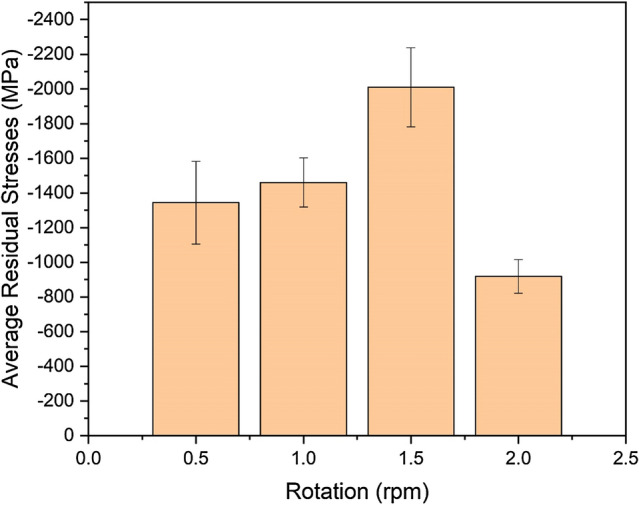
Residual stress evolution in the CrN/Cr_1-x_Al_x_N multilayers deposited onto gas nitrided diesel piston rings as a function of the applied carousel rotational speed.

Figure 11 displays how the carousel rotation influences the mean residual stresses in the coatings. Compression rises until it reaches 1.5 rpm, then decreases. Increasing carousel rotation exposes the growing coating more often to the ion bombardment, but the exposure to the ion beam becomes shorter. This implies that faster carousel rotations initially enhance the implantation of knock-on ions and lower the occurrence of stress-relieving mechanisms, such as collision cascades, atomic rearrangements, and the formation of residual vacancies within the growing coating. These effects corroborate the increasing coating residual stresses between 0.5 and 1.5 rpm. When the carousel rotates too fast at 2.0 rpm, the stress accumulation owing to the ion implantation surpasses an upper limit, thus leading to detrimental effects, such as the formation of extended lattice imperfections and three-dimensional voiding and micro-cracking, that also relieve stresses.

The impact of carousel rotational speed on the surface roughness could be evaluated with the aid of 3D AFM surface maps. Figure 4c illustrates the 3D AFM surface map acquired from the top surface of the CrN/Cr_1-x_Al_x_N multilayer manufactured at − 150 V and 2.0 rpm. Figure 4b displays how the carrousel rotational speed influences the coating surface roughness. The resulting Ra-values were 93.5 nm, 84.8 nm, 83.2 nm, and 178.2 nm for 0.5 rpm, 1.0 rpm, 1.5 rpm, and 2.0 rpm, respectively. This steep increase of Ra for too high rotational speeds (i.e., tiny periodicities) reveals that when the layer thickness deposited after each individual carousel rotation becomes very thin (in the present work, it reaches approximately 4.6 nm for 2.0 rpm), the heterogeneity of the deposited atomic layer thicknesses enhances the coating surface roughness.

**Figure 12:**
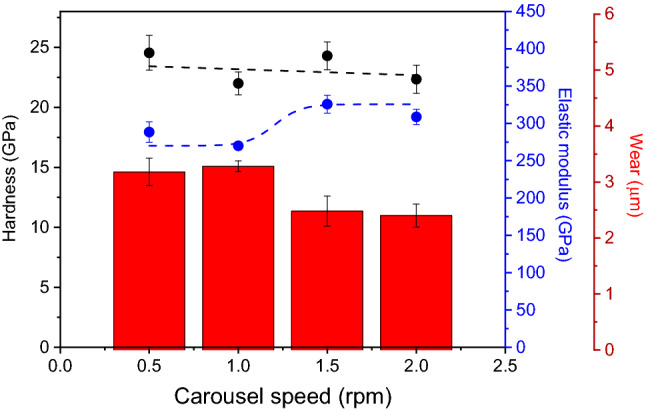
Hardness, elastic modulus, and wear depth against the cylinder liner measured for CrN/Cr_1-x_Al_x_N multilayers grown on diesel piston rings at a constant bias (− 150 V) with different periodicities produced by variable carousel rotational speed (0.5 rpm, 1.0 rpm, 1.5 rpm, and 2.0 rpm).

Figure 12 compares hardness, elastic modulus, and wear against the cylinder liner for CrN/Cr_1-x_Al_x_N multilayers deposited onto gas nitrided diesel piston rings at a constant bias (− 150 V) with distinct periodicities. The hardness values do not change significantly, and the average value is close to 23 GPa. This finding indicates that the range of periodicities generated here for the multilayers is around the maximum promoted by the superlattice effect. In addition, the residual stress behavior produced by different carousel rotational speeds (Fig. 11) does not significantly influence the hardness of the coatings, thus indicating that the effect of the multilayer periodicity prevails over the compressive residual stresses. This is likely due to the presence of high compression in the GPa-range after all coating procedures investigated here. The elastic modulus exhibits a sigmoidal behavior with respect to the carousel rotational speed (i.e., periodicity). Reduced elastic modulus E_r_ starts at lower values (~ 275 GPa for 0.5 rpm), then increases and reaches a plateau at 325 GPa for 2.0 rpm. The most promising wear results for use in piston rings are encountered for multilayers manufactured at -150 V with 1.5 rpm and 2.0 rpm (~ 2.5 µm wear depth). Both elastic modulus and wear resistance confirm the major influence of nanometric multilayer periodicities on the mechanical and tribological behavior of the coatings.

**Figure 13:**
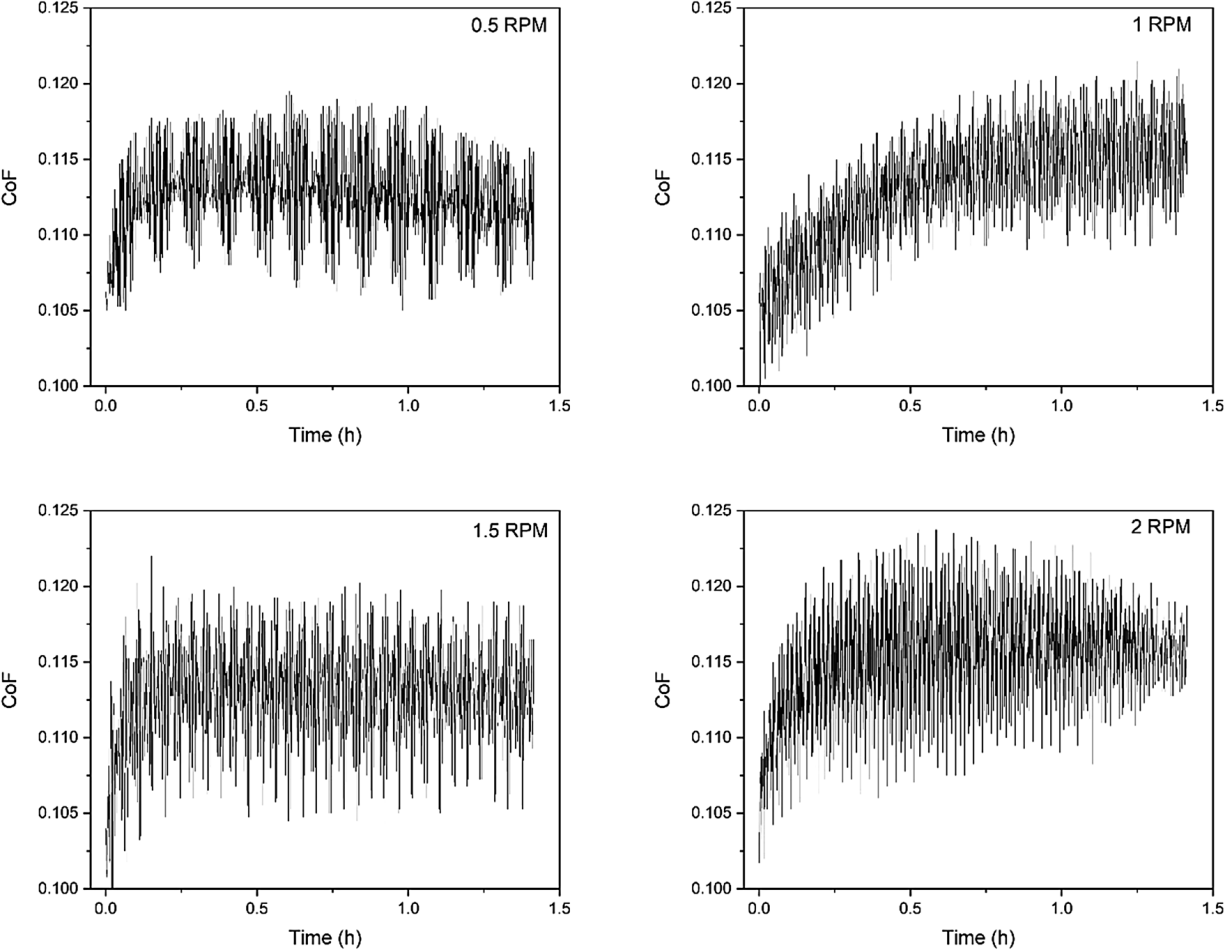
CoF evolution during wear tests for the multilayer coatings produced using different carousel rotation speeds.

The variation of CoF along the wear tests for the coatings produced under different rotational speeds is presented in Fig. 13. Although the samples deposited using 0.5 and 1 rpm showed a less steep increase in CoF along time and stabilized in lower values (about 0.115 CoF) in comparison with 1.5 and 2 rpm, all coatings presented CoF correspondent to state-of-the-art CrN-containing coatings in the literature and therefore are candidates for piston ring applications^45–49^.

In addition, the mechanical and tribological properties of the CrN/Cr_1-x_Al_x_N multilayers sputtered at a constant bias (− 150 V) with distinct periodicities by varying the carousel rotational speed (0.5 rpm, 1.0 rpm, 1.5 rpm, and 2.0 rpm) were compared with state-of-the-art values measured for commercial PVD coatings of CrN applied to diesel piston rings^5^, see Table 2. Both the commercial CrN coatings and the multilayers developed in this work were sputtered onto the outer diameter surface of diesel piston rings. Moreover, it is important to emphasize that the commercial CrN coatings were manufactured by cathodic arc evaporation. In contrast, the CrN/Cr_1-x_Al_x_N multilayers were obtained using an innovative hybrid magnetron sputtering process characterized in terms of sputtering power by 33% of HiPIMS and 67% of dcMS. Regarding H and E, and considering the most promising features (see Table 2), the innovative approach proposed here yields superior mechanical strength and wear resistance (36% and 23%, respectively) when compared to commercial PVD coatings of CrN while maintaining comparable average COF values.

**Table 2:** Summary of H, E, wear against the cylinder liner, and H^3^/Er^2^ values obtained for CrN/Cr_1-x_Al_x_N multilayers manufactured at a constant bias of − 150 V and different rotational speeds of the carrousel.

Property	Carousel rotational speed (rpm)/periodicity (nm)				CrN (commercial)
	0.5/17.5	1.0/7.0	1.5/6.7	2.0/4.6	
H (GPa)	24.6 ± 1.4	22.0 ± 1.0	24.3 ± 1.2	22.4 ± 1.2	11.8–15.7
E (GPa)	288.3 ± 13.8	270.0 ± 5.2	325.7 ± 11.9	308.7 ± 10.3	250
Wear depth (µm)	3.18 ± 0.24	3.28 ± 0.10	2.48 ± 0.27	2.40 ± 0.21	5^5^
H^3^/Er^2^ (GPa)	0.18	0.15	0.15	0.12	0.026–0.06
Average COF	0.113 ± 0.003	0.114 ± 0.003	0.113 ± 0.003	0.115 ± 0.003	< 0.15^5^

The data of commercial CrN is included for comparison.

**Figure 14:**
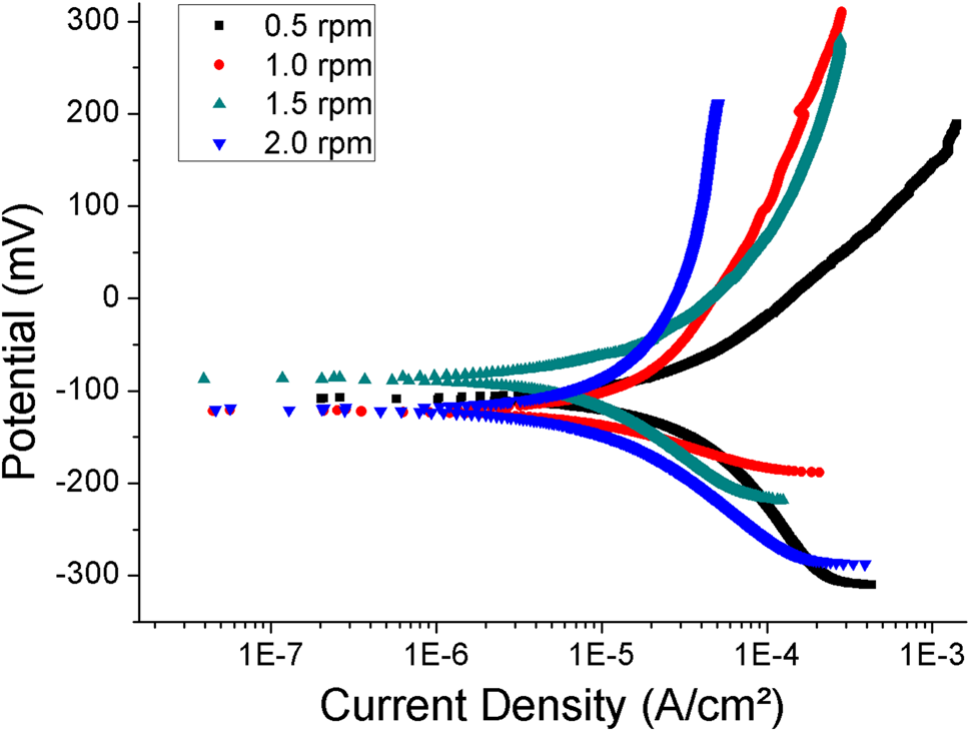
Potentiodynamic polarization curves obtained in 3.5 wt.% NaCl solution for CrN/Cr_1-x_Al_x_N multilayers with distinct periodicities produced at the constant bias of − 150 V and by varying the rotational speed of the carousel.

The corrosion resistance of the CrN/Cr_1-x_Al_x_N multilayers with distinct periodicities can be inferred from potentiodynamic polarization curves obtained in 3.5 wt.% NaCl solution, as shown in Fig. 14^53^. NaCl solution is used to analyze the impact of the environmental working conditions of the piston rings. The values of corrosion potential (E_corr_) and corrosion current (I_corr_) for the different multilayers are summarized in Table 3. The values of corrosion current are within the range expected for CrN obtained by different methods^54^.

**Table 3:** Values of E_corr_ and I_corr_ obtained in the potentiodynamic polarization tests for the multilayers produced with different periodicities.

	Carrousel rotational speed (rpm)/periodicity (nm)			
	0.5/17.5	1.0/7.0	1.5/6.7	2.0/4.6
E_corr_ (mV)	− 108.1	− 120.1	− 86.8	− 120.1
I_corr_ (µA/cm^2^)	38.1	23.4	13.7	20.7

The most notable trend is the systematic decrease in anodic current density for the coatings produced at increasing rotational speeds, i.e., decreasing periodicities. This trend indicates increased polarization resistance, which can be attributed to the higher electrical resistance of the coating and/or the formation of a thin passive film. However, considering that the chemical composition across the multilayers is identical, the different electrochemical response is then attributed to the microstructural effect. The thicknesses of the coating, average crystallite size, and presence of imperfections contribute to the electrochemical response. The lowest anodic current density is verified for the multilayer produced at 1.5 rpm (6.7 nm periodicity), which is of similar total thickness and apparent grain size as the coating produced at 0.5 rpm (17.5 nm periodicity), see Fig.7a,d. This observation corroborates the results of periodicities smaller than 10 nm resulting in denser and less defective coatings. For increased periodicities obtained under 1.0 and 0.5 rpm, higher gradients of growth stresses are expected to build up along the columnar grains within the multilayer. The tiny periodicities obtained under 2.0 rpm introduce severe interatomic disorder via grain boundaries and interfaces. Both effects contribute to a deterioration of the corrosion resistance and are consistent with the observed electrochemical response.

Finally, it should be considered that the most relevant industrial target of a coating is its lifetime, in particular for innovative piston rings to be applied in low emission combustion engines, where inspection and maintenance are difficult. This lifetime is mainly determined by the trade-off between wear resistance, corrosion, and localized plastic deformation (H^3^/Er^2^). Under this viewpoint, a tailored profile is achieved by the CrN/Cr_1-x_Al_x_N multilayer sputtered at − 150 V and 1.5 rpm with a periodicity of 6.7 nm.

## Conclusions

CrN/Cr_1-x_Al_x_N superlattices were manufactured by an innovative hybrid magnetron sputtering process (HiPIMS/dcMS), capable of yielding more competitive deposition rates for sputtering techniques. The structures were deposited onto gas-nitrided diesel piston rings made of AISI 440 martensitic stainless steel. Process design was conducted in two cycles to identify the optimum bias level and multilayer periodicity without influencing the chemical modulation within the multilayers. For potential use in piston rings of low emission combustion engines, a trade-off combination of technological properties was identified considering hardness, elastic modulus, resistance to plastic deformation (H^3^/Er^2^), wear against the cylinder liner and corrosion resistance in 3.5 wt.% NaCl solution.

The results demonstrated that independent of the bias level, the CrN/Cr_1-x_Al_x_N superlattices grow with strong (100) fiber orientation in the normal direction of the coating surface. Surface roughness was enhanced when the deposited atomic layers became very thin and heterogeneous after each carousel rotation, i.e., for too small periodicities and elevated carousel rotational speeds. The maximum hardness promoted by the superlattice effect was verified in the range of 5 to 15 nm periodicity for CrN/Cr_1-x_Al_x_N multilayers. Hardness, wear and corrosion resistances demonstrated a more relevant influence of the multilayer periodicity since elevated compressive stresses in the GPa-range were generated anyway in all deposition routes investigated here. The resistance to plastic deformation, as indicated by H^3^/Er^2^, appeared though to be the most adequate mechanical property to correlate with the engineering surface properties, such as wear and corrosion, when carrying out materials selection and coating design for piston rings. The wear depth produced in all CrN/Cr_1-x_Al_x_N multilayers by reciprocating sliding against the cylinder liner was 50% lower than in the commercial CrN reference, thus indicating that the multilayers grown by the hybrid sputtering process proposed here is a suitable solution for piston rings of the next generation of diesel combustion engines.

## Materials and methods

### Coating deposition

**Figure 15:**
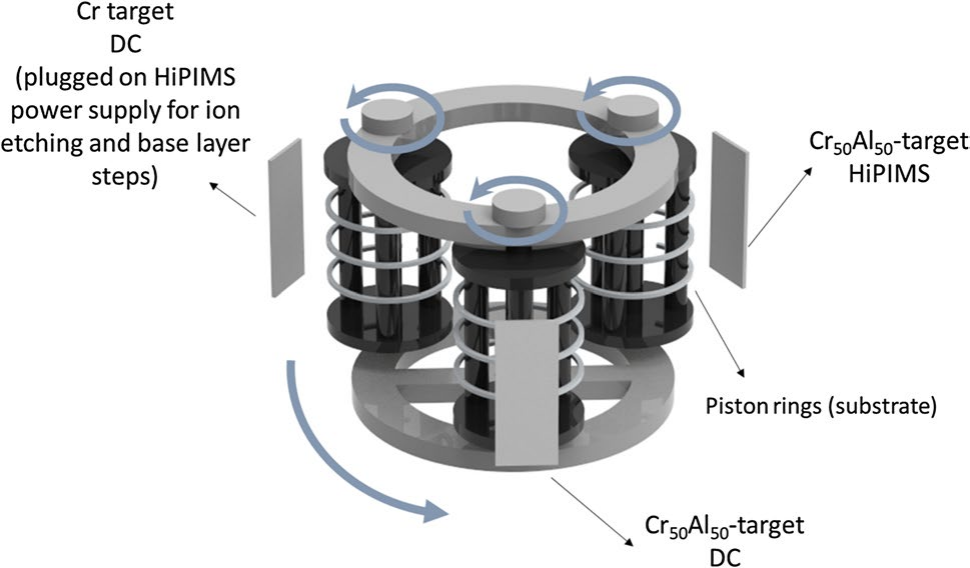
Scheme of the carousel rotation within the sputtering system mounted with gas-nitrided piston rings and three sputtering targets: two of a CrAl alloy (50–50 at.%) and one of pure Cr.

The sputtering system used in this research consists of a HiPIMS TruPlasma 4004 (TRUMPF, Germany) and two DC MDX Pinnacle (Advanced Energy, USA) power supplies installed in a Plasma-HiPIMS-250 chamber (Plasma LIITS, Brazil), and an additional MDX Pinnacle power supply used for substrate biasing. A pure Cr and two CrAl (50–50 at.%) alloy targets (214 mm × 106 mm each), all with a purity of 99.5%, were employed for coating deposition. The targets were connected to the power supplies in the following manner: a CrAl alloy to HiPIMS, another CrAl alloy to DC, and the pure Cr to DC, as displayed in Fig. 15.

CrN/Cr_1-x_Al_x_N multilayers were sputtered onto gas-nitrided diesel piston rings made of AISI 440 martensitic stainless steel with a nitrided layer of approximately 60 µm thickness and hardness varying from 850 and 750 HV0.1. The piston rings were provided by MAHLE Metal Leve, Brazil, and had an outer diameter of 131 mm, an inner diameter of 126 mm, and a thickness of 3.5 mm. Prior to coating deposition, the piston ring surfaces were subjected to a two-step pre-processing under pure argon plasma (50 sccm). First, ion etching with Cr^+^ ions was conducted for 1 h using the HiPIMS process at a mean power density of 3.0 W/cm^2^, 100 Hz and 50 µs t_on_, as well as a substrate bias of − 800 V to enable physical surface cleaning and shallow ion implantation. Then, the deposition of a base layer of metallic chromium followed, using the same substrate bias as that applied later during the multilayer deposition.

The CrN/Cr_1-x_Al_x_N multilayers were grown under an argon/nitrogen plasma (at 40/50 sccm, respectively), applying substrate bias of either − 120 V, − 150 V, or − 180 V. The periodicity of the multilayers was modulated by changing the carousel rotational speeds (0.5 rpm, 1.0 rpm, 1.5 rpm or 2.0 rpm). All other parameters were kept constant, in particular: mean power density of 4.5 W/cm^2^ and peak HiPIMS power density of 90 W/cm^2^ applied to the targets with peak current density over the target area of 0.2027 A/cm^2^, working pressure of 0.266 Pa, a substrate temperature of 400 °C, HiPIMS frequency of 500 Hz, HiPIMS t_on_ of 200 µs and substrate to target distance of 65 mm. These parameters were identified as optimal in another study described by Guimaraes et al.^28^.

### Coating characterization

The morphology and microstructure of the coatings were evaluated using a Field Emission Scanning Electron Microscope F-50, FEG-SEM (FEI, The Netherlands). Both top surface and fractured cross-section were inspected. An Atomic Force Microscope NanoSurf Flex, AFM (NanoSurf, Switzerland) was used to map and quantify the surface topography generated by each deposition strategy.

Depth profile analysis by glow discharge optical emission spectroscopy (GDOES) was applied to evaluate the chemical composition within the CrN/Cr_1-x_Al_x_N multilayers. The depth profiles were determined on a multilayer deposited onto a flat AISI 304L stainless steel substrate that was included in the PVD chamber along with the piston rings. GDOES was carried out in DC excitation mode (constant voltage-constant current mode) using a Spectruma Analytik GmbH GDA 750 HR spectrometer equipped with a 2.5 mm diameter anode. The depth profiles were verified by triplicate using glows obtained in argon atmosphere (5.0 purity) with average discharge pressure of 5·10–2 hPa. The excitation parameters were set to 1000 V and 12 mA, and the sputtering rate was calculated so that the measuring depth was at least 75 µm. The WinGDOES software was applied to automatically determine quantitative depth profiles of mass concentration.

X-ray diffraction (XRD) was applied to phase identification, texture analysis, and to determine the periodicity of the multilayers. All the XRD analyses were conducted in an X-Ray diffractometer Rotaflex Ru200B (Rigaku, Japan) equipped with a copper rotatory anode (Kα − 1.5418 Å) and operating at an accelerating voltage of 40 kV and a current of 60 mA. The θ–2θ diffraction geometry was used for a 2θ range from 35° to 85°, using a step size of 0.05° in the 2θ-scale, and acquisition time of 5 s per step. The phases were identified using the software High Score Plus (PANalytical, Holland) and by matching data with the ICSD database. Texture analysis was conducted by determining the texture coefficient (${T}_{hkl}$) for each (hkl) reflection using Eq. (1), where the absorption factor (${A}_{\uptheta /2\uptheta }$) is given by Eq. (2). Im and IICDD correspond to the measured and the powder reference peak intensities, respectively, whereas t is the coating thickness and µ the linear attenuation coefficient (1215 cm^−1^ for Cu radiation and CrN coatings).

1 $$T_{{hkl}} = \frac{{{\raise0.7ex\hbox{${I_{{hkl}}^{m} }$} \!\mathord{\left/ {\vphantom {{I_{{hkl}}^{m} } {A_{{\theta /2\theta }} {\text{ (}}\theta _{h} {\text{)}}}}}\right.\kern-\nulldelimiterspace} \!\lower0.7ex\hbox{${A_{{\theta /2\theta }} {\text{ (}}\theta _{h} {\text{)}}}$}}}}{{I_{{hkl}}^{{ICDD}} }} \cdot \frac{{\sum\nolimits_{{hkl}} {I_{{hkl}}^{{ICDD}} } }}{{{\raise0.7ex\hbox{${\sum\nolimits_{h} {I_{{hkl}}^{m} } }$} \!\mathord{\left/ {\vphantom {{\sum\nolimits_{h} {I_{{hkl}}^{m} } } {A_{{\theta /2\theta }} {\text{ (}}\theta _{h} {\text{)}}}}}\right.\kern-\nulldelimiterspace} \!\lower0.7ex\hbox{${A_{{\theta /2\theta }} {\text{ (}}\theta _{h} {\text{)}}}$}}}}$$

2 $${A}_{\uptheta /2\uptheta }\left({\uptheta }_{hkl}\right)=1-{e}^{-\frac{2\mu t}{\mathrm{sin}\theta }}$$

The periodicity ($\Lambda )$ of the multilayers was determined based on the occurrence of satellite peaks surrounding a major diffraction line due to the formation of multilayers with coherent superlattices by using Eq. (3), where m and n are the order of the satellite peaks, ${\uptheta }_{m}$ and ${\uptheta }_{n}$ their peak positions and λ the X-ray wavelength.

3 $${\Lambda }_{XRD} = \frac{\left|m-n\right|\uplambda }{2\left|\mathrm{sin}\left({\uptheta }_{m}\right)-\mathrm{sin}({\uptheta }_{n})\right|}$$

The periodicity of the multilayers was also verified, taking into account the experimental parameters of the multilayer deposition: the total coating thickness, deposition time, and rotational speed of the piston ring surface (i.e., including carousel rotation and planetary self-rotation of the ring surfaces), according to Eq. (4):

4 $${\Lambda }_{*}=\frac{thickness\left[\upmu m\right]}{time \left[hours\right]*speed \left[rpm\right]*60}$$

X-ray stress analyses were carried out with a Mo-Kα lab source to determine the mean (CrN + CrAlN) residual coating stresses using the sin^2^ψ technique applied to the (440) and (424 + 600) diffraction lines of the fcc-CrN phase. Seven ψ-tilts equally spaced in the sin^2^ψ-scale were employed up to sin^2^ψ of 0.9. The diffraction elastic constants (DEC) were calculated using the Eshelby-Kroener approach^36^ using the single crystal elastic constants for CrN^37^.

Instrumented nanohardness measurements were carried out using a PB1000 Mechanical Tester (Nanovea, USA). Each experiment consisted of 15 indentations (5 × 3 matrix) carried out on the top of the coating by applying a maximum load of 30 mN at a loading rate of 60 mN/min and producing indentations 50 µm apart from each other. The penetration depth was kept below one-tenth of the coating thickness to ensure no interference of substrate deformation.

Wear bench tests were conducted against a gray cast iron cylinder liner under reciprocating conditions at 130 °C, a normal load of 360 N, a frequency of 900 rpm, and 0W20 oil as a lubricating agent enriched with Al_2_O_3_ particles of 0.3 µm grain size at a concentration of 5 g/L. The Coefficient of Friction (CoF) of each test was calculated.

Corrosion tests were conducted using a potentiostat–galvanostat VersaSTAT4 (Princeton Applied Research, USA) connected to a three-electrode set-up and a 3.5 wt.% NaCl solution as electrolyte. The exposed area of the working electrode was 0.76 cm^2^, Ag,AgCl/KCl electrode was used as reference (0.197 V vs. SHE), and a solid platinum electrode as the counter electrode. Prior to polarization, the open-circuit potential (OCP) was monitored for 1200 s. Afterward, a potentiodynamic scan was measured from − 0.35 V to + 0.35 V vs. OCP at the scan rate of 0.167 mV/s. The corrosion currents were determined by Tafel extrapolating the anodic and cathodic slopes to the corrosion potential^53^.

## Data Availability

The data that support the findings of this study are available from the author, P.R.T.A., upon reasonable request.
